# Weight loss and adherence to postoperative follow-up after vertical gastrectomy for obesity treatment

**DOI:** 10.1590/ACB360203

**Published:** 2021-02-22

**Authors:** Roclides Castro de Lima, Thállisso Martins da Silva Rodrigues, Christian Lamar Scheibe, Giuliano Peixoto Campelo, Luís Eduardo Veras Pinto, Gustavo José Cavalcante Valadão, Gustavo Pereira Câmara de Carvalho, Marcos Roberto Dias Machado, José Aparecido Valadão, Patrícia Cavalcante Ribeiro de Lima, Plinio da Cunha Leal, Caio Marcio Barros de Oliveira, Ed Carlos Rey Moura

**Affiliations:** 1 Fellow Master degree. Universidade Federal de São Paulo – Postgraduate Program in Interdisciplinary Surgical Science – São Paulo (SP), Brazil.; 2 Graduate student. Universidade Federal do Maranhão – Medicine Course – São Luís (MA), Brazil.; 3 MD. Hospital São Domingos – Surgery Department – São Luís (MA), Brazil.; 4 MD. Universidade Federal do Maranhão – Hospital São Domingos – Clinical Department – São Luís (MA), Brazil.; 5 Fellow PhD degree. Universidade Federal de São Paulo – Postgraduate Program in Interdisciplinary Surgical Science – São Paulo (SP), and Assistant Professor. Universidade Federal do Maranhão – Department of Medicine – São Luís (MA), Brazil.

**Keywords:** Bariatric Surgery, Gastrectomy, Obesity

## Abstract

**Purpose:**

To analyze the effectiveness of vertical gastrectomy in the treatment of obese patients, adherence to clinical follow-up and the influence of factors such as gender and age.

**Methods:**

This is a retrospective, observational and descriptive study, conducted with patients undergoing vertical gastrectomy, operated at Hospital São Domingos, between January 2016 and July 2018.

**Results:**

Most patients undergoing vertical gastrectomy were female (n = 193, 72.28%) and had a mean age of37.11 ± 8.96 years old. The loss of follow-up was 56.18%. Among adherent patients (n = 117; 43.82%), most patients were female (n = 89; 76.07%) and had a mean age of 37.92 ± 9.85 years old. The mean body mass index (BMI) of the adherents in the preoperative was 37.85 ± 3.72 kg/m^2^. Both BMI and excess weight (EW) showed a statistically significant difference between pre- and postoperative period. Percentage of excess weight loss (% EWL) was satisfactory for 96.6% of adherent patients. Older patients had a statistically significant lower % EWL compared to the other groups.

**Conclusions:**

Vertical gastrectomy was effective in the treatment of obese patients, with significant weight loss.

## Introduction

Obesity is defined as a chronic disease characterized by a high accumulation of fat in the body, culminating in cardiometabolic disorders^1^^,^ 2. Due to its high incidence in several countries, it is referred to as a worldwide epidemic and it is categorized as an endocrine, nutritional and metabolic disease^2^.

According to WHO data, world figures corresponding to obesity tripled from 1975 to 2016, and projections for 2025 point out to approximately 1 billion obese adults worldwide^3^^,^ 4. Data have shown that 50% of the adult Brazilian population is overweight and 19.8% is considered specifically obese^2^.

Obesity has a complex and multidisciplinary treatment, and the main line to be followed is the improvement of eating habits and practice of physical activities; however, this strategy fails in 95% of patients^2^^,^ 5.

In light of the struggles to lose weight, bariatric surgery proves to be an effective treatment measure, as it provides a progressive and sustained weight loss^6^^,^ 7.

Vertical gastrectomy consists of removing 80% of the greater curvature of the stomach, mainly the body and bottom, without altering the anatomy of the small intestine. Many studies suggest that vertical gastrectomy provides an excess weight loss of 50% to 70% within 5 years after surgery^2^^,^ 7.

Some methods are used to detect the patient’s nutritional status and define obesity criteria, such as the body mass index (BMI), which considers obese individuals that have a BMI > 30^8^. The percentage of excess weight loss (% EWL) is a parameter that is used to assess the patient’s weight loss after surgery, as a manner to assess the presence or absence of treatment effectiveness (% EWL > 50%). The formula is given by the percentage difference of weight loss in relation to EW^9^.

Given the above-mentioned, this study aims to carry out an analysis about the effectiveness of vertical gastrectomy as an intervention method in the treatment of obese patients followed up on for a 1-year period, as well as adherence to clinical follow-up in the postoperative period and the influence of factors such as gender and age on this parameter.

## Methods

### Ethical aspects

There was no intervention of any kind between the researchers and the patients included in the study. Therefore, the Informed Consent Term was waived. The project was approved by the ethics and research committee of Hospital São Domingos and platform Brazil with the registration CAAE 52819216.5.0000.5085.

### Study design

This is a retrospective, observational and descriptive study.

### Inclusion criteria

Patients undergoing vertical gastrectomy operated at Hospital São Domingos in São Luís, state of Maranhão, Brazil, between January 2016 and July 2018, aged between 18 and 65 years and with a minimum follow-up of 1 year.

### Exclusion criteria

Patients undergoing another surgical technique or revision surgery. Patients who missed the follow-up.

### Data collection and analyzed variables

A protocol form was used to collect and record information that was collected from data from surgical reports, appointments, and electronic medical records.

In order to assess patients’ outpatient adherence, a follow-up consultation in the postoperative period at the Bariatric Surgery Service was investigated after a minimum period of 1 year and a maximum period of 1 year and 2 incomplete months.

The following formulas were used to assess the BMI, EW and % EWL:

- Body mass index (BMI, in kg/m^2^): obtained from body weight in kilograms divided by height in squared meters;
- Excess weight in surgery (EW, in kg): the difference between presurgery weight and optimum weight;
- Excess weight loss (% EWL): percentage difference of weight loss in relation to EW, which was used as an indicator for successful surgery.

### Statistical analysis

For statistical calculation, the software GraphPad Prism 6 (GraphPad Software, Inc) was used, assuming a significance level of 0.05. The measures of central trend were described for gender, age, body mass index, EW and % EWL. Body mass index and EW were calculated using the Wilcoxon test (p < 0.05). Excess weight loss was performed using the Mann–Whitney test (p < 0.05). The comparison of the % EWL by age group was performed via analysis of variance (ANOVA) followed by Tukey’s range test as a post-test (p < 0.05). The assumptions of normality complied with the D’Agostino and the Shapiro–Wilk tests.

## Results

Two hundred sixty-seven medical records were analyzed. As for the population make-up of this study, 72.28% of the patients are female and 27.72% are male. The mean age of the patients was 37.11 ± 8.96 years, with a minimum of 18 years and a maximum of 65 years. In the assessment of outpatient adherence, of the 267 patients, only 117 (43.82%) adhered to the follow-up. Of these, 76.07% of the patients were female and 23.93% were male. The mean age of these patients was 37.92 ± 9.85 years, with a minimum of 18 years and a maximum of 65 years. The median weight in the preoperative period was 101.80 ± 15.26 kg, with a minimum of 75 kg and a maximum of 160 kg. The median weight in the postoperative period of patients was 70.61 ± 13.47 kg, with a minimum of 46 kg and a maximum of 110 kg (Table 1).

**Table 1:** Adherent and nonadherent to the 1-year follow-up after vertical gastrectomy. Characterization of adherent patients in relation to age, pre- and postsurgical weight.

Variable				General		Female		Male
Nonadherent				150		193 (72.28%)		74 (27.72%)
Adherent				117 (43.82%)		89 (76.07%)		28 (23.93%)
(Mean ± Standard Deviation)		Age (years)		37.92 ± 9.8		37.38 ±9.16		39.64 ± 11.82
		Weight presurgery (kg)		101.80 ± 15.26		97.22 ±11.92		116.35 ± 15.77
		Weight postsurgery (kg)		70.61 ± 13.47		66.14 ± 10.99		84.82 ± 10.53

The mean BMI found preoperatively was 37.85 ± 3.72 kg/m^2^. In females, the mean BMI corresponded to 37.63 ± 3.55 kg/m**^2^** and in males, 38.56 ± 4.21 kg/m^2^. The mean BMI found in the postoperative period was 26.58 ± 3.69 kg/m^2^. In females, the mean BMI corresponded to 26.01 ± 3.62 kg/m^2^ and, in males, 28.38 ± 3.38 kg/m^2^. There was a statistically significant difference between the pre- and postoperative BMI for both the female and male group and for the general group. The mean overweight prior to and after vertical gastrectomy showed a statistically significant difference. The mean EW in the preoperative period was 35.54 ± 10.76 kg, statistically higher than that one found after 1 year of vertical gastrectomy, which corresponded to 4.35 ± 9.86 kg (Table 2).

**Table 2:** Comparisons between mean BMI and EW before and after vertical gastrectomy.

Variable				Before (mean ± SD)		After (mean ± SD)		p-value*
BMI (kg/m^2^)		General		37.85 ± 3.72		26.58 ± 3.69		0.0001
		Female		37.63 ± 3.55		26.01 ± 3.62		0.0001
		Male		38.56 ± 4.21		28.38 ± 3.38		0.0001
Weight excess (kg)		General		35.54 ± 10.76		4.35 ± 9.86		0.0001

* Wilcoxon test.

**Figure 1:**
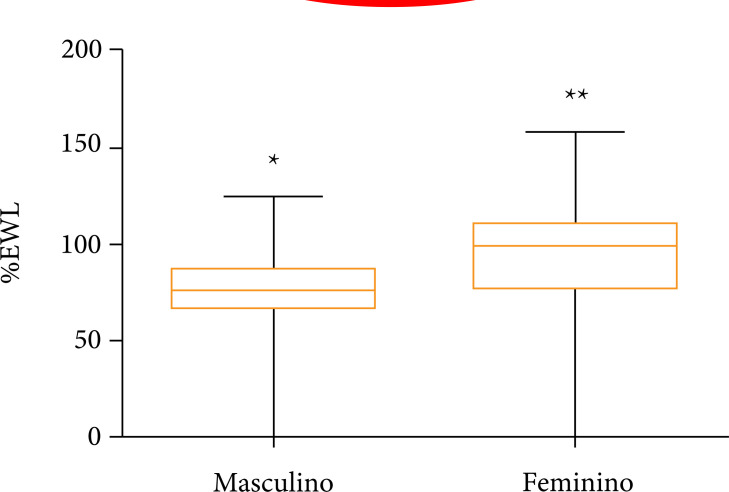
Comparison of measures of central trend of % EWL after 1 year of vertical gastrectomy between genders (male and female groups) (p < 0.05). P < 0.05 denoted by different symbols (* and **) between the experimental groups (Mann–Whitney test).

The mean % EWL in the evaluated patients was 90.86 ± 27.53%, with a minimum value of 0% and a maximum of 158.10%. Among the adherent patients who returned to the outpatient clinic, 113 patients (96.6%) of the patients reached the goal (% EWL > 50%). Among the patients who did not reach the goal, there were 3 women and 1 man. Analyzing only the male gender, the mean found was 76.98 ± 22.50%, whereas in the female group the mean corresponded to 95.24 ± 27.63%. Thus, a statistically significant difference was found when compared to the % EWL in relation to gender, with the female gender having % EWL statistically higher than the male group (Fig. 1 and Table 3).

**Table 3:** Comparison among the means of % EWL as per gender and age group.

	Gender									p-value*
Excess weight lost (%)	Female (n = 89; 76%)				Male (n = 28; 24%)					0.0006
mean ± SD	95.24 ± 27.63 %				76.98 ± 22.50 %					
	**Age group (years)**									p-value*
Excess weight lost (%)		< 30 (n = 20; 17.1%)		30 to < 40 (n = 55; 47%)		40 to < 50 (n = 26; 22.3%)		> 50 (n = 16; 13.6%)		0.0139
mean ± SD		100.10 ± 26.44		92.02 ± 25.31		93.24 ± 21.85		71.57 ±36.76		

* Mann–Whitney test; **Analysis of variance–ANOVA followed by the Tukey’s test.

**Figure 2:**
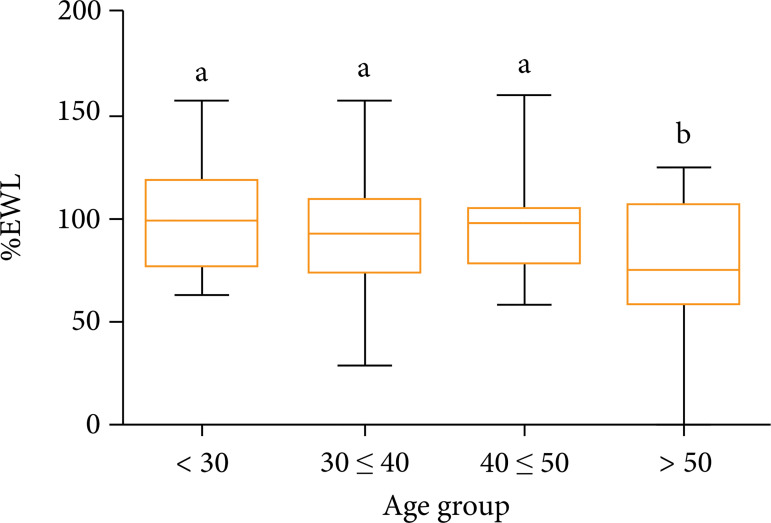
Comparison of means of % EWL among different age groups (years) (p < 0.05). P < 0.05 denoted by different letters (a, b) among the experimental groups (analysis of variance–ANOVA followed by the Tukey’s test).

The analysis of the mean % EWL between 4 different age groups (< 30 years, 30–39 years, 40–49 years > 50 years) through analysis of variance (ANOVA–Tukey) showed a statistical difference between one of the groups in relation to the others. Patients older than 50 years had a statistically lower % EWL compared to groups in the other three age groups analyzed (p = 0.013). The groups with patients aged less than 30 years, aged between 30 and 39 years, and aged between 40 and 49 years did not present significant differences in the % EWL among them. The mean % EWL of patients under the age of 30 was 100.10 ± 26.44%, among patients aged 30 to 39 years the mean was 92.02 ± 25.31%, patients aged between 40 and 49 years the mean was 93.24 ± 21.85%, finally, the mean % EWL among the patients older than 50 years was 71.57 ± 36.76% (Fig. 2 and Table 3).

## Discussion

Obesity is a global pandemic and results in major comorbidities^10^. The most successful long-term strategy continues to be bariatric surgery, which allows patients with losses already described between 50 and 75% of EW^11^.

Some studies have shown that vertical gastrectomy is associated with greater weight loss than some other bariatric treatments (clinical pharmacological therapy, intragastric balloon or gastric banding) and that vertical gastrectomy results are as good in the medium term as those achieved by gastric bypass^12^^–^ 14. The benefits of vertical gastrectomy over Roux-en-Y gastric bypass (RYGB) include the preservation of endoscopic access to the digestive tract, a reduction in the sensation of hunger by removing the gastric fundus (the main site of ghrelin production), significant remission of comorbidities and very low rates of morbidity and mortality in the postoperative period^12^^–^ 14. The results of vertical gastrectomy were so convincing that it has become an independent procedure, achieving an increase in procedures both nationally and internationally^14^.

Postoperative adherence provides benefits to patients not only for the prevention of long-term complications but also for sustained weight loss. Jennings *et al*., in 2013, noticed greater weight loss in patients who had outpatient adherence^15^^,^ 16.

In this study, among the adherent patients, most were female, maintaining a similar percentage in relation to the total number of women assessed in the survey.

As for the age variable, no association was found regarding patients missing follow-ups, as in the studies by Compher *et al*.^17^, and Belo *et al*.^18^. However, a study pointed out that there is greater adherence follow-up from patients aged 40 to 59 years, as they are more engaged, have greater stability at work and private health plans^19^.

Bodyweight both pre- and postoperatively was higher in men. According to Jones *et al.* 20, the fact is justified as men have a higher percentage of lean mass than women and are taller than women.

Both BMI and EW showed a statistically significant difference between pre- and post-surgical after 1 year of vertical gastrectomy, corroborating the study by Jaruvongvanich *et al.* 7, showing the effectiveness of vertical gastrectomy.

The % EWL greater than 50% is considered adequate. In this study, weight loss was satisfactory for 96.6% of patients, with an overall average of 90.86%. Boza *et al.* 21 obtained a EWL of 88%; they presented values for % EWL below those ones found in this study. For Obeidat and Shanti^22^, the high initial weight loss is a positive predictor for a good subsequent loss (how long it is considered initial weight loss) – 6 months.

There was a greater loss of % EWL in women, with a statistically significant difference. In turn, Coleman and cols showed that women had a higher % EWL than men in the long term, but his study also included patients who underwent Roux-en-Y gastric bypass^23^.

Older than 50 years patients had a statistically significant lower % EWL compared to other groups. These results are similar to some studies^24^^–^ 26. This fact can be explained by the low metabolic rate in the elderly, decreased oxidation of fat in the elderly and weakened lipolytic activity in postmenopausal women who make up the majority of the group, in addition to lower levels of physical activity performed in this age group^27^.

## Conclusions

Vertical gastrectomy has evolved a lot over time and it is now a safe procedure with consistent and sustainable results, allowing the reduction of obesity and related diseases, providing a huge health benefit that directly impacts the patient’s life quality. Preoperative preparation and awareness are important, especially in patients with a higher risk of missing the follow-up (men) and less response (> 50 years).
